# Three new species and reassessment of the rare Neotropical ant genus *Leptanilloides* (Hymenoptera, Formicidae, Leptanilloidinae)

**DOI:** 10.3897/zookeys.133.1479

**Published:** 2011-10-05

**Authors:** Marek L. Borowiec, John T. Longino

**Affiliations:** 1 Department of Entomology, One Shields Avenue, University of California at Davis, Davis, California 95616, USA; 2 Department of Biology, University of Utah, Salt Lake City, Utah 84112, USA

**Keywords:** dorylomorphs, doryline section, army ants, taxonomy, systematics, metatibial gland, morphology

## Abstract

We describe three new species of the Neotropical ant genus *Leptanilloides*: *Leptanilloides gracilis* **sp. n.** based on workers from Mexico and Guatemala, *Leptanilloides erinys* **sp. n.** based on workers and a gyne from Ecuador, and *Leptanilloides femoralis* **sp. n.** based on workers from Venezuela. The description of *Leptanilloides gracilis* is a northern extension of the known range of the genus, now numbering eleven described species. We also describe and discuss three unassociated male morphotypes from Central America. We report the occurrence of a metatibial gland in *Leptanilloides* and a fused promesonotal connection (suture) in some species. We provide a modified, detailed diagnosis of the genus and a revised key to the worker caste of the known species.

## Introduction

*Leptanilloides* Mann, 1923 is a genus of rarely collected Neotropical ants with army ant-like habits, convergently similar to the Old World genus *Leptanilla* Emery, 1870. Little is known about their biology (Brandão et al. 1999b), but a number of papers on taxonomy and phylogenetic affinities has been published. In 1923 Mann described *Leptanilloides biconstricta* from Bolivia and placed it in the subfamily Dorylinae (Mann 1923). Subsequently the genus was considered a member of the Cerapachyinae (Brown 1975, Bolton 1990a, 1990b) and then placed in its own subfamily Leptanilloidinae (Baroni Urbani et al. 1992, Bolton 1994). Brandão et al. (1999a) revised the subfamily, adding the new genus *Asphinctanilloides* Brandão et al., 1999, describing three new species of *Leptanilloides*, and proposing a morphology-based phylogeny. Bolton (2003) provided a detailed taxonomic history of the genus and subfamily. Longino (2003) and Donoso et al. (2006) described further species, the latter also providing information on hitherto unknown gyne and male castes. Ward (2007) used molecular data to associate a male from Costa Rica with workers described by Longino (2003). Also with the aid of molecular methods, Ward and Brady (2009) established that the male-based genus *Amyrmex* Kusnezov, 1953, previously placed in Dolichoderinae, is in fact a member of Leptanilloidinae and a potential senior synonym of *Asphinctanilloides*. There is no doubt that Leptanilloidinae represents a group within a larger clade of the so called dorylomorph ants, as evidenced by a multitude of morphological (Bolton 1990b, Brady and Ward 2005) and molecular (Brady 2003, Moreau et al. 2006, Brady et al. 2006, Ward and Brady 2009) data. The genus level taxonomy, however, is still unsettled, and names are expected to change in the future due to the unresolved affinity of *Amyrmex* (Ward & Brady 2009) and the uncertain distinction between *Leptanilloides* and *Asphinctanilloides* (Longino 2003, Donoso et al. 2006, Ward and Brady 2009). New species will also undoubtedly continue to be discovered, as the ratio of distinct species to collecting events remains high.

Below we describe three new species, mostly collected by leaf litter sifting and extraction with the Winkler apparatus, with the exception of the type series of *Leptanilloides erinys*, where workers were initially collected from the forest floor by sifting and later extensive search revealed an entire colony. The newly described species are incorporated into a key to all the known species of *Leptanilloides*. In addition we describe three male morphotypes from Central America. These are males from Malaise trap samples and thus unassociated with workers. We expect future molecular work to associate males and workers, although we hypothesize the association of one of the male morphotypes with *Leptanilloides gracilis* based on geographic overlap. We also provide evidence for the occurrence of metatibial glands in *Leptanilloides*, discuss the structure of the promesonotal suture, and give a detailed diagnosis of the genus based on the worker caste.

## Methods

Measurements were made using a Wild M5A stereomicroscope at 50× magnifications with a dual-axis Nikon micrometer wired to a digital readout. Color photographs were prepared using a Leica MZ 16 stereomicroscope with a JVC digital video camera. The scanning electron micrographs were prepared using a Zeiss/LEO 1450VP SEM at the California Academy of Sciences. All images were processed using Syncroscopy Automontage and Zerene Systems Zerene Stacker software and cleaned and adjusted using Adobe Photoshop.

The description of wing venation is based on an unpublished scheme by Bolton (pers. comm.), including description of veins as tubular, nebulous, and spectral. Recommendations for illustration of veins follow Mason (1986). For male genitalia, we adopt the terminology of Yoshimura and Fisher (2011). All specimen data along with images have been deposited on the AntWeb public database (http://www.antweb.org/).

In lists of material examined and other reporting of specimen data an error term may occur after latitude and longitude values. This error term is the sum of GPS error and spatial extent of the sampling area around the point where latitude and longitude were recorded.

The following measurements and indices are used:

**HW** head width: maximum width in full face view. HW for males includes eyes (workers are eyeless).

**HL** head length: maximum length along midline in full face view, measured medially from the anteriormost part of the head (anterior edge of frontal lobes) to the center of posterior margin. Excavation of posterior head margin reduces HL.

**SL** scape length: maximum length measured without condyle and neck.

**LAII, LAIII, LAIV, LAXIII** (male only): length of second, third, fourth and terminal (13th) antennal segments, respectively.

**EL** eye length (male only): measured in full face view, maximum length of eye parallel to midline.

**MH** mesosoma height: in lateral view, maximum height measured from the lowermost point of mesopleuron (in front of middle coxa) to dorsal edge of mesosoma, measured perpendicular to long axis of mesosoma.

**ML** mesosoma length: in lateral view, maximum longitudinal distance from farthest point on anterior face of pronotum, excluding the neck, to posteroventral corner of mesosoma.

**PrW** pronotal width: maximum width in dorsal view.

**PW** petiole width: maximum width of abdominal segment II in dorsal view.

**PL** petiole length: maximum length of abdominal segment II in dorsal view, measuring only the length of the petiolar posttergite.

**AIIIW** third abdominal tergite width: maximum width in dorsal view.

**AIIIL** third abdominal tergite length: maximum length in dorsal view measured medially, measuring only the length of the posttergite, excluding pretergite III (helcium).

**AIVW** fourth abdominal tergite width: maximum width in dorsal view.

**AIVL** fourth abdominal tergite length: maximum length in dorsal view measured medially, excluding pretergite.

**FFeW** front femur width: maximum width in lateral view.

All leg measurements below are taken as maximum length measured along extensor (outer) surface:

**FFeL** fore femur length.

**HFeL** hind femur length.

**HTiL** hind tibia length.

**CI** cephalic index: HW/HL×100.

**MI** mesosomal index: MH/ML×100.

**PI** petiolar index: PW/PL×100.

All measurements are given in mm.

Depositories

**AMNH** American Museum of Natural History, New York, NY, USA.

**BMNH** Natural History Museum, London, United Kingdom.

**CASC** California Academy of Sciences, San Francisco, CA, USA.

**EAPZ** Escuela Agricola Panamericana, Tegucigalpa, Honduras.

**ECOSCE** Colección Entomológica de El Colegio de la Frontera Sur, Unidad San Cristóbal, Chiapas, Mexico.

**FMNH** Field Museum of Natural History, Chicago, IL, USA.

**LACM** Los Angeles County Museum of Natural History, CA, USA.

**MCZC** Museum of Comparative Zoology, Harvard University, Cambridge, MA, USA.

**MIZA** Museo del Instituto de Zoologia Agricola, Universidad Central de Venezuela, Maracay, Venezuela.

**MLBC** Marek Borowiec personal collection, Davis, CA, USA.

**MZSP** Museu de Zoologia da Universidade de São Paulo, São Paulo, Brazil.

**NMNH** Smithsonian Institution, National Museum of Natural History, Washington, DC, USA.

**QCAZ** Museo de Zoología de la Pontifícia Universidad Católica del Ecuador, Quito, Ecuador.

**UCDC** The Bohart Museum of Entomology, University of California, Davis, CA, USA.

**UVGC** Colleción de Artrópodos, Universidad del Valle de Guatemala, Guatemala City, Guatemala.

## Results

During our study we have had a chance to examine type material of most species of Leptanilloidinae and carry out a detailed SEM study of *Leptanilloides erinys*, *Leptanilloides gracilis*, *Leptanilloides femoralis* and *Leptanilloides nubecula*. We have found that, contrary to previous studies (Brandão et al. 1999a, Longino 2003, Donoso et al. 2006), at least some species of *Leptanilloides* do possess a metatibial gland and have the promesonotal connection fused and immobile.

**Figure 1.**
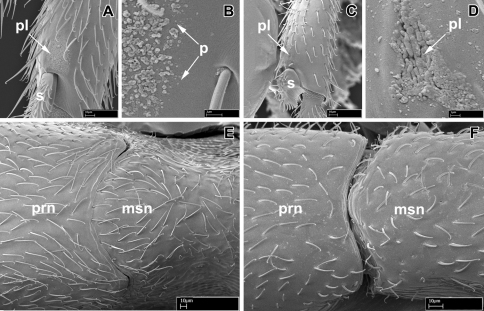
Scanning electron micrographs of selected characters. **A–B, E** *Leptanilloides nubecula* worker (CASENT0234587) **A** apex of hind tibia showing metatibial gland pore plate (*pl*) and tibial spur (*s*) **B** pore plate of metatibial gland showing pores (*p*) **C–D** *Leptanilloides erinys* worker (CASENT0234584) **C** apex of hind tibia showing metatibial gland pore plate (*pl*) and tibial spur (*s*) **D** pore plate (*pl*) of metatibial gland magnified **E** pronotum (*prn*) and mesonotum (*msn*) in dorsal view showing partly fused promesonotal connection **F** *Leptanilloides gracilis* (CASENT0234585) pronotum (*prn*) and mesonotum (*msn*) in dorsal view showing complete promesonotal connection.

The metatibial gland was first recognized and considered a synapomorphy of dorylomorph ants (=doryline section) by Bolton (1990b) and subsequently described in detail by Hölldobler et al. (1996). It has been claimed to be absent from hitherto described *Leptanilloides* (Brandão et al. 1999a, Longino 2003, Donoso et al. 2006). With the aid of SEM we have been able to observe small differentiated patches of porous cuticle and granulate secretion on the hind tibia of *Leptanilloides erinys* and *Leptanilloides nubecula* (Figures 1A–D). We believe these represent vestigial pore plates of the metatibial gland. It is possible other species of *Leptanilloides* possess it, although due to positioning of legs in our specimens we were unable to find it in *Leptanilloides gracilis* and *Leptanilloides femoralis*. Since the pore plate is extremely small and *Leptanilloides* ants themselves are tiny, the gland is impossible to discern with a stereomicroscope under magnifications of about 100×. Also, due to its position on the flexor (inner, ventral) surface of the tibia, it is easily overlooked even under SEM.

The promesonotal connection has also been described as universally unfused and flexible in workers of the genus (Brandão et al. 1999a, Longino 2003, Donoso et al. 2006). We have found that this character is in fact very variable in *Leptanilloides*, ranging from completely unfused and apparently flexible in *Leptanilloides biconstricta*, *Leptanilloides caracola*, *Leptanilloides erinys*, *Leptanilloides femoralis*, *Leptanilloides gracilis*, *Leptanilloides improvisa* and *Leptanilloides sculpturata* (Figure 1F) and gradually increasing in fusion in *Leptanilloides legionaria* through *Leptanilloides mckennae* to *Leptanilloides nubecula* and *Leptanilloides nomada* (Figure 1E), where the connection seems to be completely fused dorsally, barely visible as a faint groove. The fusion of the promesonotal connection correlates with other morphological features: the lateroclypeal teeth are reduced, abdominal segment III is small in relation to segment IV, and the spiracles of abdominal segment III are shifted posteriorly. The latter three characters had already been noticed by Donoso et al. (2006) and interpreted as blurring the distinction between *Asphinctanilloides* and *Leptanilloides*. The segregation of *Leptanilloides* into two natural species groups seems to be supported by molecular data, although taxon sampling is still unsatisfactory (Phil Ward, pers. comm.). Adding somewhat intermediate species to the dataset, like *Leptanilloides legionaria* that has a small abdominal segment III but only weakly fused promesonotal suture, or *Leptanilloides biconstricta* with apparently complete promesonotal connection but intermediate abdominal segment III, may blur this distinction.

Given the new morphological findings, coupled with comparative character investigation (Marek Borowiec, unpublished) for other dorylomorph lineages, we feel it useful to provide a detailed and revised definition of *Leptanilloides* based on the worker caste.

The known leptanilloidine males show substantial variation (see Donoso et al. 2006, Ward 2007, Ward and Brady 2009, descriptions below) and the lack of definite worker-male associations prevents us from characterizing the male caste of the genus in a structured way. Ward and Brady (2009) enumerated differences between the known *Amyrmex* morphotypes and the then known males of *Leptanilloides*, *mckennae* and *nubecula*, pointing out that the distinction is weak and that undescribed Leptanilloidinae material weakens it even further. If the three Central American males described here turn out to belong to *Leptanilloides*, then for the following characters the distinction is weakened further still: small body size of Leptanilloidinae male 1 (HW on average < 0.30), short scapes of Leptanilloidinae male 3 (SL/LAII 1.4–1.9), relatively short legs of the Central American males (HTiL/HL 1.2–1.4), parameres shorter than petiole in male 3, veins M and Cu diverging at cu-a in all the below described morphotypes, and the absence of free abscissae of M joining R in Leptanilloidinae male 3. The only character from their list differentiating *Amyrmex* from *Leptanilloides* that seems to hold is the more narrow and elongate submarginal cell of fore wing in the former.

### Diagnosis of Leptanilloides based on worker caste

Antennae with 12 segments.

Apical antennal segment slender, not swollen; round in cross-section.

Clypeus with well developed, translucent lamella (apron).

Lateroclypeal teeth (same as “genal” teeth in Donoso et al. 2006) present or absent.

Parafrontal ridges absent or weakly developed.

Preocular grooves absent.

Frontal carinae vertical, very reduced and fused, completely exposing antennal sockets.

Antennal scrobes absent.

Maxillary palps two-segmented, except in *gracilis*, where apparently weakly fused and forming one segment; labial palps two-segmented (palp formula 1,2 or 2,2) (in situ count in *gracilis*, *femoralis* and *legionaria*, also reported by Brandão et al. 1999a).

Mandibles subtriangular, edentate or with small, blunt teeth on both masticatory and basal margins.

Eyes absent.

Ocelli absent.

True occipital margin concealed behind vertex in full face view.

Ventrolateral margins of head with carina encircling the foramen only.

Head ventrally with carina complete around foramen magnum, evenly rounded.

Pronotal flange not separated from collar by distinct ridge.

Promesonotal connection complete and apparently flexible (*biconstricta*, *caracola*, *erinys*, *femoralis*, *gracilis*, *improvisa*, *sculpturata*) or partly to almost completely fused and not flexible (*legionaria*, *mckennae*, *nomada*, *nubecula*).

Propleura and mesopleura distinctly separated, the connection continuous with promesonotal portion.

Mesometapleural sulcus usually visible, weakly impressed and running towards metanotal sulcus, anepisternum not delineated dorsally or posteriorly.

Transverse mesopleural sulcus absent.

Posterior head, mesosoma, petiole and abdominal segment III immarginate.

Petiole laterally above spiracle immarginate.

Petiole anterodorsally immarginate.

Helcium narrow, posterior face of petiole and anterior face of abdominal segment III well-developed.

Abdominal segment III smaller or much smaller than succeeding segment IV, which is constricted at the presegmental portion.

Abdominal segment III anterodorsally immarginate.

Abdominal segment IV not conspicuously the largest segment.

Abdominal tergite IV not folding over sternite, and anterior portions of sternite and tergite are equally well visible in lateral view.

Cinctus of abdominal segment IV simple, not cross-ribbed.

Girdling constriction posterior to abdominal segment IV present on sternites V and VI

Abdominal tergite VII (pygidium) reduced and short, unarmed.

Abdominal sternite VII (hypopygium) unarmed.

Mid tibia with one simple spur, hind tibia with single pectinate spur (most species) or both tibiae with two simple spurs (only *gracilis*) (spur formula 1s,1p or 2s,2s).

Middle and hind basitarsus not widening distally, circular in cross-section.

Posterior flange of hind coxa not produced as raised lamella.

Metatibial gland present, reduced (observed in *erinys* and *nubecula*).

Metabasitarsal sulcus absent.

Pretarsal claws simple.

Although originally the genus *Asphinctanilloides* had been differentiated from *Leptanilloides* by several characters (Brandão et al. 1999a), subsequent descriptions of new taxa somewhat blurred this distinction. At present at least presence of a deep metanotal groove and absence of a constriction between abdominal pre- and postsegments V and VI can be regarded synapomorphic for *Asphinctanilloides*. Ward and Brady (2009) discussed the subject in detail and noted that differentiation of *Asphinctanilloides* may render *Leptanilloides* paraphyletic.

### Key to workers of Leptanilloides

**Table:** 

1	Abdominal segment III (postpetiole) in lateral view much smaller than adjoining fourth abdominal segment (Figure 2A). Spiracle of segment III shifted posteriad on anteromedian side of tergite (Figure 2A). Body size relatively large, HL 0.68–0.75	2
–	Abdominal segment III in lateral view nearly as high as abdominal segment IV (Figure 2B–D). Spiracle of segment III situated forward on the tergite (Figures 2B–D). Body size relatively small, HL 0.31–0.50	5
2	Head subquadrate, CI 85–88; lateral margins nearly straight and parallel (Figure 2E). Propodeal declivity short and vertical, propodeum with dorsal and posterior faces clearly differentiated (Figure 2A) (Ecuador)	*Leptanilloides nomada*
–	Head subrectangular, CI 75–83; lateral margins convex (Figure 2F). Propodeal declivity usually rounded without clear distinction between dorsal and posterior face (cf. Figure 2B, Figure 4 in Donoso et al. 2006)	3
3	Head sculpture less dense, at most 10–12 shallow foveolae across face at midlength. Lateral margins of the head distinctly convex. Posterior margin of the head slightly concave (Figure 2F) (Colombia)	*Leptanilloides legionaria*
–	Head sculpture more dense, with at least 15 foveolae across face at midlength. Lateral margins of the head slightly convex. Posterior margin of the head deeply concave. (cf. Figure 2F, Figure 3 in Donoso et al. 2006)	4
4	Legs shorter, HW/HTiL×100 > 78. Hypostomal teeth present (Figure 2H) (Ecuador)	*Leptanilloides nubecula*
–	Legs longer, HW/HTiL×100 < 78. Hypostomal teeth absent (Figure 2I) (Costa Rica)	*Leptanilloides mckennae*
5	Lateroclypeal teeth absent. Masticatory margin of mandibles edentate (Figure 5 in Donoso et al. 2006) (Ecuador)	*Leptanilloides caracola*
–	Lateroclypeal teeth present. Masticatory margin of mandibles with teeth (Figure 4D)	6
6	In lateral view, sternite of abdominal segment III (postpetiole) distinctly bulging anteriorly, making the sternal portion of the segment deeper than tergite (Figure 2D). Abdominal segment IV narrowly attached to the preceding segment III, and broadly to succeeding segment V, so that there is a contrast between widths of anterior and posterior articulation of the segment IV in lateral view (Colombia, Bolivia)	*Leptanilloides biconstricta*
–	In lateral view, sternite of abdominal segment III rather evenly rounded, and making the sternal and tergal portions subequal (Figures 2B, 2C). Abdominal segment IV relatively broadly attached to the preceding segment III, so that there is little contrast between widths of anterior and posterior articulations of segment IV in lateral view (Figures 4E, 5E)	7
7	In lateral view, petiolar sternite distinctly bulging medially (Figure 2C)	8
–	In lateral view, petiolar sternite bulging anteriorly (Figures 2B, 2D)	9
8	Hind tibia with two very small, simple spurs, without pectinate spur clearly visible under 50× magnification (Figure 2J). Petiolar spiracle opening in an excavation distinctly larger than propodeal spiracle (Figure 5G, 5H). Flange over the metapleural gland opening sharply pointed posteriorly (Figure 5G) (Mexico, Guatemala)	*Leptanilloides gracilis*
–	Hind tibia with a large pectinate spur, clearly discernable under 50× magnification. Petiolar spiracle not in excavation, similar and subequal to or smaller in diameter than propodeal spiracle (Figures 4G, 4H). Flange over metapleural gland opening rounded posteriorly (Figure 4G) (Venezuela)	*Leptanilloides femorali*s
9	Smaller species, HW < 0.30. Slender with narrow head, CI < 70 (Figure 2G) (Colombia)	*Leptanilloides sculpturata*
–	Larger species, HW > 0.30. Head broader, CI > 70	10
10	Size larger, HW = 0.38 on single known specimen. Petiolar sternite conspicuously excavated anteroventrally in lateral view (Figure 2B). Flange over metapleural gland opening rounded posteriorly (Figure 22 in Brandão et al. 1999a) (Ecuador)	*Leptanilloides improvisa*
–	Smaller, HW < 0.35. Petiolar sternite not conspicuously excavated anteroventrally in lateral view (Figure 3E, 3J). Flange over metapleural gland opening sharply pointed posteriorly (Figure 3J) (Ecuador)	*Leptanilloides erinys*

#### Leptanilloides erinys sp. n.

urn:lsid:zoobank.org:act:AACFA7BE-4A9E-4B85-8FD4-A35686BC8FCE

http://species-id.net/wiki/Leptanilloides_erinys

Figures 1C–D 3A–L

##### Type material.

 Holotype worker: ECUADOR, *Napo*: Yanayacu Biological Station, −0.60°, −77.88°, 2200m, secondary cloud forest, 9 December 2009 (*M. L. Borowiec* #MLB091209.01) [unique specimen identifier CASENT0234603] [QCAZ]. Paratype gyne and workers: about a hundred specimens with the same data as holotype, point-mounted and in alcohol [AMNH, BMNH, CASC, FMNH, LACM, MCZC, MZSP, NMNH, QCAZ, UCDC].

*Non-type material examined*: 6 workers with the same data as holotype, except collection date 1 December 2009, sifted leaf litter (*M. L. Borowiec* #MLB091209.04) [MLBC].

*Worker measurements* (*holotype*): HW 0.31, HL 0.41, SL 0.20, MH 0.19, ML 0.50, PrW 0.22, PW 0.11, PL 0.15, AIIIW 0.20, AIIIL 0.16, AIVW 0.33, AIVL 0.24, FFeW 0.09, FFeL 0.23, HFeL 0.22, HTiL 0.26, CI 76, PI 75, MI 38.

*Worker measurements and indices* (*11 measured*): HW 0.31–0.32, HL 0.41–0.43, SL 0.19–0.21, MH 0.18–0.20, ML 0.49–0.53, PrW 0.20–0.23, PW 0.12–0.14, PL 0.15–0.17, AIIIW 0.19–0.22, AIIIL 0.14–0.18, AIVW 0.31–0.33, AIVL 0.21–0.25, FFeW 0.09–0.10, FFeL 0.23–0.25, HFeL 0.23–0.24, HTiL 0.26–0.28, CI 74–78, PI 74–82, MI 36–41.

##### Diagnosis.

Worker can be distinguished by combination of relatively small size, promesonotal articulation complete and articulated, abdominal segment III large relative to petiole, presence of lateroclypeal teeth, relatively heavy sculpturing, parafrontal ridges absent, flange overhanging metapleural gland opening pointed posteriorly. It is most similar to *Leptanilloides sculpturata* from Colombia, but can be distinguished by significantly larger size (HW ≥0.31 in *erinys* versus 0.20–0.26 in *sculpturata*), relatively broader head (CI >70 vs. 58–67) and shorter petiole (PI >74 vs. PI=70 measured in holotype). *Leptanilloides erinys* also differs in weaker sculpturation of head dorsum, with small foveolae separated by about their diameter (Figure 1G), while in *Leptanilloides sculpturata* the foveolae are separated by much less than their diameter, often contiguous (Figure 7 in Brandão et al. 1999a).

##### Worker description.

 With characters of *Leptanilloides* (see Diagnosis of *Leptanilloides* based on worker caste, above). Head elongate and subquadrate with lateral margins nearly straight and parallel. Posterior corners rounded and posterior border weakly concave. Parafrontal ridge absent. Clypeus laterally with blunt tooth pointing outwards. Mandible short, masticatory margin with three distinct blunt teeth basally and basal margin crenulate. Basal and masticatory margin distinct, but separated by a rounded angle. Palp formula unknown. Scape short and clavate. Antennal joints submoniliform, gradually increasing in size toward apex but not forming an antennal club. Mesosoma long, slender and flattened. Pronotum with a flexible promesonotal suture. Metanotal groove absent. Propodeum unarmed. Propodeal declivity very short and rounding into the dorsal face. Propodeal spiracle round, situated posteriorly on the sclerite. Metapleural gland flange conspicuous, translucent and posteriorly pointed. Femur not conspicuously enlarged, relatively slender. Mid tibia with one simple and hind tibia with one pectinate spur. Metatibial gland absent. Petiole smaller than abdominal segment III (postpetiole) in dorsal view. Petiole rectangular, uniformly wide across its length in dorsal view and with straight sides and abdominal segment III dilating posteriorly. In lateral view, petiolar tergite posteriorly sloping, without well differentiated posterior face and without long tubulated portion posteriorly. Petiolar sternite bulging anteriorly. Abdominal sternite III evenly rounded. Metasoma relatively robust. Abdominal segments IV–VI subequal in length in dorsal view and separated by strong constrictions. Segment VII (pygidium) small and mostly concealed by the preceding segment, U-shaped.

Head with abundant deep punctures and smooth interspaces on average about equal to puncture diameter, except on sides where punctures sparser, separated by more than their diameter. Mesosoma and abdomen more finely and sparsely punctate. Laterally on lower pronotum, entire mesopleuron, propodeum and petiole fine microreticulate sculpture present. Head, body and appendages with abundant, rather coarse, short and erect hairs. Body color yellowish to brownish.

*Gyne measurements and indices* (*1 measured*): HW 0.41, HL 0.46, SL 0.20, MH 0.24, ML 0.63, PrW 0.25, PW 0.24, PL 0.20, AIIIW 0.40, AIIIL 0.23, AIVW 0.45, AIVL 0.35, FFeW 0.11, FFeL 0.26, HFeL 0.27, HTiL 0.30, CI 88, PI 118, MI 38.

##### Gyne description.

 Subdichthadiigyne. Head rectangular, lateral borders weakly convex and posterior border distinctly concave. Compound eyes present and comprised of about ten weakly defined ommatidia, situated behind head midlength. Mandible subtriangular, masticatory margin crenulate, basal margin edentate. Clypeal apron present, small. Wingless, without any wing sclerites or wing buds. Petiole enlarged, taller than in worker and wider than long in dorsal view. Abdominal segment III broadly attached to following segments, tergosternal fusion not assessed. Petiolar and abdominal segment III spiracles located as in workers. Girdling constriction of abdominal segments IV–VI weakly developed and conspicuous only on segment IV. Tergite of abdominal segment VII (pygidium) large, not U-shaped and mostly concealed by preceding segment as in workers. Promesonotal connection present, articulated. Entire body covered with dense pubescence, more erect than in worker.

##### Male.

 unknown.

**Figure 2.**
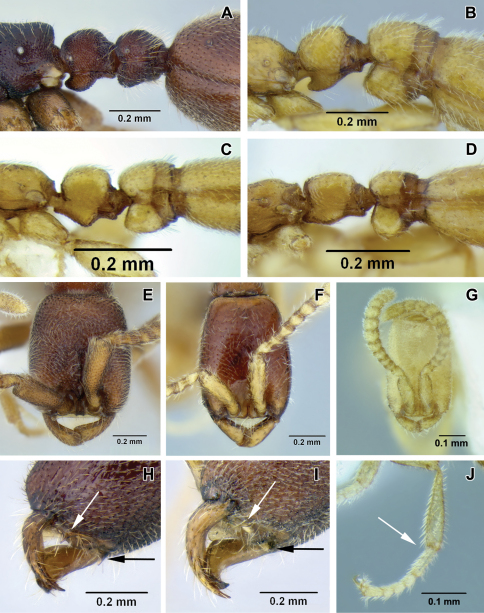
**A–D** lateral view focusing on propodeum, petiole and abdominal segment III **A** *Leptanilloides nomada* worker (CASENT0234620) **B** *Leptanilloides improvisa* holotype worker (MCZ type 35284) **C** *Leptanilloides femoralis* holotype worker (CASENT0106180) **D** *Leptanilloides biconstricta* paralectotype worker (NMNH type 25705) **E–G** head in full-face view **E** *Leptanilloides nomada* worker (CASENT0234620) **F** *Leptanilloides legionaria* worker (CASENT0234619) **G** *Leptanilloides sculpturata* holotype worker (USNM ENT 00533059) **H, I** ventrolateral view of head capsule focusing on hypostoma **H** *Leptanilloides nubecula* worker (CASENT0234621) **I** *Leptanilloides mckennae* paratype worker (INBIOCRI001281144) **J** hind leg of *Leptanilloides gracilis* worker (CASENT0612940).

**Figure 3.**
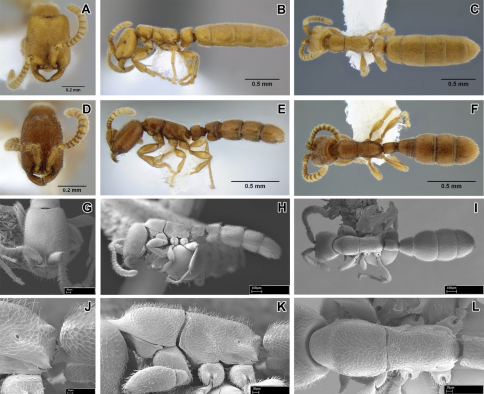
*Leptanilloides erinys*, new species **A–C** paratype gyne (CASENT0234616) **A** head in full-face view, B: body in lateral view **C** body in dorsal view. **D–F** paratype worker (CASENT0234596) **D** head in full-face view **E** body in lateral view **F** body in dorsal view **G–L** paratype worker (CASENT0234584) **G** head in full-face view **H** body in lateral view **I** body in dorsal view **J** propodeum and anterior petiole in lateral view **K** mesosoma in lateral view **L** mesosoma in dorsal view.

##### Biology.

 This species was collected in montane cloud forest habitat. Workers were first located in sifted leaf litter. After scraping leaf litter and removing root mat in an area of about 3m^2^, a colony was discovered ca. 5cm below ground in a single soil cavity adjacent to a root. In a mass of workers a single gyne could be seen, as well as many slender larvae. The gyne did not have an extended gaster, there were no eggs visible in the nest, and all the larvae were of approximately the same size, suggesting synchronized brood production.

#### Leptanilloides femoralis sp. n.

urn:lsid:zoobank.org:act:17523937-4A7E-4C76-8E33-5067E5089300

http://species-id.net/wiki/Leptanilloides_femoralis

Figures 2C 4A–I

##### Type material.

 Holotype worker: VENEZUELA, *Aragua*: Pico Periquito, PN Henri Pittier, 10.339°, −67.706°, 1500m, sifted litter (leaf mold, rotten wood) 17 August 2008 (*P. S. Ward* #16198.06) [unique specimen identifier CASENT0106180] [MIZA]. Paratype workers: 22 workers with the same data as holotype, point-mounted and in alcohol [AMNH, BMNH, CASC, FMNH, LACM, MCZC, MIZA, MZSP, NMNH, QCAZ, UCDC].

*Worker measurements* (*holotype*): HW 0.25, HL 0.32, SL 0.14, MH 0.12, ML 0.42, PrW 0.15, PW 0.09, PL 0.12, AIIIW 0.13, AIIIL 0.11, AIVW 0.22, AIVL 0.18, FFeW 0.09, FFeL 0.19, HFeL 0.19, HTiL 0.22, CI 78, PI 75, MI 29.

*Measurements in mm and indices* (*7 measured*): HW 0.23–0.25, HL 0.32–0.34, SL 0.14–0.16, MH 0.12–0.14, ML 0.41–0.44, PrW 0.15–0.17, PW 0.08–0.10, PL 0.12, AIIIW 0.12–0.14, AIIIL 0.11–0.14, AIVW 0.22–0.23, AIVL 0.17–0.19, FFeW 0.08–0.09, FFeL 0.18–0.19, HFeL 0.19–0.20, HTiL 0.20–0.22, CI 71–78, PI 67–80, MI 29–32.

##### Diagnosis.

 Worker relatively slender and small compared to most species in the genus, promesonotal connection complete and articulated, abdominal segment III (postpetiole) large relative to petiole, lateroclypeal teeth present, sculpturing moderate, parafrontal ridges present, flange overhanging metapleural gland opening rounded posteriorly. In general habitus and size it is most similar to *Leptanilloides gracilis* but can be distinguished by the small opening of petiolar spiracle (situated in large depression in *gracilis*), the pointed flange over the metapleural gland (rounded in *gracilis*), single pectinate spur on hind tibia (two simple spurs in *gracilis*), and relatively broader femur (FFeW 0.08–0.09 in *femoralis*, 0.06–0.07 in *gracilis*). Both *femoralis* and *gracilis* are similar to *biconstricta* from Bolivia and *improvisa* from Ecuador, but can be distinguished by the distinctly bulging sternite of the petiole, with the bulge most prominent medially (versus indistinctly broadened anteriorly in *biconstricta* and *improvisa*).

##### Worker description.

 With characters of *Leptanilloides* (see Diagnosis of *Leptanilloides* based on worker caste, above). Head elongate and rectangular with lateral margins nearly straight and parallel. Posterior corners rounded and posterior border concave. Parafrontal ridge distinct. Clypeus laterally with blunt tooth distinctly pointing outwards. Mandible short, masticatory margin with small teeth and basal margin crenulate. Basal and masticatory margins distinct, but separated by a rounded angle. Maxillary palp two-segmented. Labial palp two-segmented (in situ count). Scape short and clavate. Antennal joints submoniliform, gradually increasing in size toward apex but not forming an antennal club. Mesosoma long, slender and flattened. Pronotum with a flexible promesonotal suture. Metanotal groove absent. Propodeum unarmed. Propodeal declivity very short and rounding into the dorsal face. Propodeal spiracle round, situated posteriorly on the sclerite. Metapleural gland flange conspicuous, translucent and posteriorly blunt. Femur enlarged, broad. Mid tibia with one simple and hind tibia with one pectinate spur. Petiole smaller than abdominal segment III (postpetiole) in dorsal view. Petiole rectangular, uniformly wide across its length in dorsal view and with straight sides and abdominal segment III dilating posteriorly. In lateral view, petiolar tergite with differentiated anterior and posterior faces, posterior tubulated portion short. Petiolar sternite distinctly bulging medially. Abdominal sternite III evenly rounded. Metasoma long and slender. Abdominal segments IV–VI subequal in length in dorsal view and separated by strong constrictions. Pygidium small and mostly concealed by the preceding segment, U-shaped.

Head with abundant punctures with smooth interspaces on average equaling puncture diameter, except on sides where punctures sparser. Mesosoma and abdomen more finely and sparsely punctate. Laterally on mesopleuron, propodeum and petiole fine microreticulate sculpture present. Head, body and appendages with abundant, rather coarse, short and erect hairs. Body color yellowish.

##### Gyne and male.

 Unknown.

**Figure 4.**
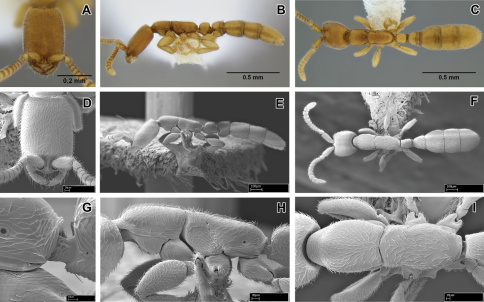
*Leptanilloides femoralis*, new species **A–C** holotype worker (CASENT0106180) **A** head in full-face view **B** body in lateral view **C** body in dorsal view **D–I** paratype worker (CASENT0234586) **D** head in full-face view **E** body in lateral view **F** body in dorsal view **G** propodeum and anterior petiole in lateral view **H** mesosoma in lateral view **I** mesosoma in dorsal view.

##### Biology.

*Leptanilloides femoralis* is known to occur in montane cloud forest habitat. The single collection was from a Winkler sample of sifted litter and rotten wood from the forest floor.

##### Discussion.

 This species is superficially very similar to *Leptanilloides gracilis* and at first sight might be considered an allopatric population of that species. However, molecular data obtained for ten nuclear gene regions from both morphotypes shows a very large amount of sequence divergence, making it extremely unlikely that the ants belong to the same species (Phil Ward, unpublished data).

#### Leptanilloides gracilis sp. n.

urn:lsid:zoobank.org:act:9D7C6CE3-5E0D-4D2B-B7AC-58CECD516225

http://species-id.net/wiki/Leptanilloides_gracilis

Figures 1F 2J 5A–I

##### Type material.

 Holotype worker: MEXICO, *Chiapas*: Sierra Morena, 16.15224°, −93.60068° ±50m, 1330m, 12 May 2008 (Project LLAMA Wa-A-01-2-22) [unique specimen identifier CASENT0234574] [MCZC]. Paratype workers: 10 workers with the same data as holotype [AMNH, BMNH, CASC, EAPZ, ECOSCE, FMNH, LACM, MZSP, NMNH, UCDC, UVGC].

*Non-type material examined*: workers: MEXICO, *Chiapas*: Sierra Morena, 16.15971°, −93.60512° ±50m, 1360m, 12 May 2008 (Project LLAMA Wa-A-01-1-24); workers: GUATEMALA, *Suchitepéquez*: 4km S Vol. Atitlán, 14.55288°, −91.19316° ±50m, 1750m, 15 June 2009 (Project LLAMA, Wa-B-09-2-43).

*Worker measurements* (*holotype*): HW 0.25, HL 0.33, SL 0.15, MH 0.15, ML 0.44, PrW 0.17, PW 0.10, PL 0.13, AIIIW 0.15, AIIIL 0.12, AIVW 0.24, AIVL 0.19, FFeW 0.07, FFeL 0.19, HFeL 0.18, HTiL 0.21, CI 77, PI 76, MI 34.

*Worker measurements* (*11 measured*): HW 0.23–0.25, HL 0.31–0.33, SL 0.14–0.15, MH 0.12–0.15, ML 0.40–0.44, PrW 0.14–0.17, PW 0.09–0.10, PL 0.12–0.14, AIIIW 0.12–0.15, AIIIL 0.11–0.13, AIVW 0.21–0.24, AIVL 0.16–0.19, FFeW 0.06–0.07, FFeL 0.17–0.19, HFeL 0.17–0.19, HTiL 0.20–0.21, CI 70–81, PI 69–79, MI 29–34.

##### Diagnosis.

 Worker relatively slender and small compared to most species in the genus, promesonotal connection complete and articulated, abdominal segment III large relative to petiole, lateroclypeal tooth present, sculpturing moderate, parafrontal ridge present, flange overhanging metapleural gland opening pointed posteriorly. *Leptanilloides gracilis* is unique in the modified petiolar spiracle, opening to a conspicuous pit larger in diameter than propodeal spiracle opening (Figure 3G), maxillary palpus with only one segment and mid and hind tibia with two simple spurs. In general habitus and size it is most similar to *Leptanilloides femoralis* but can be distinguished (in addition to traits mentioned above) by the pointed flange over the metapleural gland (blunt in *Leptanilloides femoralis*) and relatively slender femur. Both *Leptanilloides gracilis* and *Leptanilloides femoralis* are similar to *Leptanilloides biconstricta* from Bolivia and *Leptanilloides improvisa* from Ecuador, but can be distinguished by the distinctly bulging sternite of the petiole, with the bulge most prominent medially (versus indistinctly broadened anteriorly in *Leptanilloides biconstricta* and *Leptanilloides improvisa*).

##### Worker description.

 With characters of *Leptanilloides* (see Diagnosis of *Leptanilloides* based on worker caste, above). Head elongate and rectangular with lateral margins nearly straight and parallel. Posterior corners rounded and posterior border concave. Parafrontal ridge distinct. Clypeus laterally with blunt tooth distinctly pointing outwards. Mandible short, masticatory margin with small teeth and basal margin crenulate. Basal and masticatory margins distinct, but separated by a rounded angle. Maxillary palp apparently fused to form one segment, although weakly constricted and similar in length to two-segmented labial palp (in situ count). Scape short and clavate. Antennal joints submoniliform, gradually increasing in size toward apex but not forming an antennal club. Mesosoma long, slender and flattened, with a flexible promesonotal suture. Metanotal groove absent. Propodeum unarmed. Propodeal declivity very short and rounding into the dorsal face. Propodeal spiracle round, situated posteriorly on the sclerite. Metapleural gland flange conspicuous, translucent and posteriorly pointed. Femur not conspicuously enlarged, relatively slender. Mid and hind tibia each with two small and simple spurs. Metatibial gland absent. Petiolar spiracle opening to conspicuous depression, in diameter exceeding propodeal spiracle. Petiole smaller than abdominal segment III (postpetiole) in dorsal view. Petiole rectangular, uniformly wide across its length in dorsal view and with straight sides and abdominal segment III dilating posteriorly. In lateral view, petiolar tergite with differentiated anterior and posterior faces, posterior tubulated portion short. Petiolar sternite distinctly bulging medially. Abdominal sternite III evenly rounded. Metasoma long and slender. Abdominal segments IV–VI subequal in length in dorsal view and separated by strong constrictions. Pygidium small and mostly concealed by the preceding segment, U-shaped.

Head with abundant punctures with smooth interspaces on average equaling puncture diameter, except on sides where punctures sparser. Mesosoma and abdomen more finely and sparsely punctate. Laterally on mesopleuron, propodeum and petiole fine microreticulate sculpture present. Head, body and appendages with abundant, rather coarse, short and erect hairs. Body color yellowish.

##### Gyne.

 Unknown.

**Figure 5.**
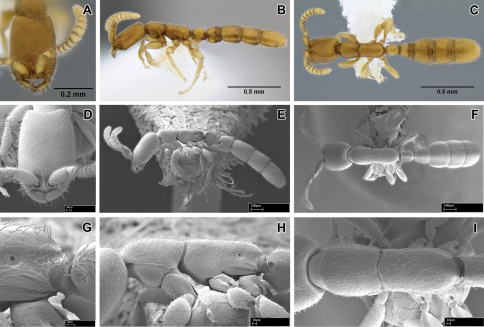
*Leptanilloides gracilis*, sp. n. **A–C** holotype worker (CASENT0234574) **A** head in full-face view **B** body in lateral view **C** body in dorsal view **D–I** paratype worker (CASENT0234585) **D** head in full-face view **E** body in lateral view **F** body in dorsal view **G** propodeum and anterior petiole in lateral view **H** mesosoma in lateral view **I** mesosoma in dorsal view.

##### Male.

 See discussion under Leptanilloidinae male 1.

##### Biology.

 The type series was collected in second growth mesophyll cloud forest. A few dozen workers were in a single “miniWinkler” sample, which is litter sifted from a 1m^2^ plot on the forest floor. Two additional workers were collected in a similar miniWinkler sample approximately 1 km distant. The species occurred in two of 100 miniWinkler samples taken at the site. The Guatemala collection was made under similar circumstances, in which a small series of workers occurred in one of 100 miniWinkler samples from a mature cloud forest habitat.

##### Discussion.

*Leptanilloides gracilis* is similar in general habitus to some other small species of the genus, especially *Leptanilloides femoralis*. It is unique, however, in some traits that may be considered autapomorphies of this species. It has the segments of the maxillary palpus fused to form one, instead of the two-segmented palpus seen in other species where the palp formula is known. The petiolar spiracle opening is situated in a conspicuous pit of diameter larger than the propodeal spiracle opening; in all other species of *Leptanilloides* the petiolar spiracle opening is simple and subequal or smaller than that of propodeal spiracle. There are two minute, simple spurs on the mid and hind tibia, while other species of the genus are known to have one simple spur on the mid tibia and a single conspicuously pectinate spur on hind tibia.

#### Leptanilloidinae male 1

Figure 6A–F

##### Material examined.

 MEXICO, *Chiapas*: 2km SE Custepec, 15.72099°, −92.95106° ±5m, 1495m, 17–24 May 2008 (LLAMA#Ma-A-02-1-02); Nahá, 16.94917°, −91.59476° ±11m, 960m, 9–13 June 2008 (LLAMA#Ma-A-07-2-02); GUATEMALA, *Izabal*: 5km NW Morales, 15.51341°, −88.86616° ±8m, 245m, 16–20 May 2009 (LLAMA#Ma-B-04-2-01); 5km NW Morales, 15.51351°, −88.86647° ±7m, 245m, 16–20 May 2009 (LLAMA#Ma-B-04-2-02); *Petén*: Cerro Cahuí, 17.00044°, −89.70346° ±5m, 140m, 22–25 May 2009 (LLAMA#Ma-B-05-1-02); Parq. Nac. Tikal, 17.24433°, −89.62201° ±6m, 270m, 22–25 May 2009 (LLAMA#Ma-B-05-2-02); 13km NW Machaquilá, 16.44491°, −89.55136° ±6m, 380m, 27–30 May 2009 (LLAMA#Ma-B-06-1-01); 13km NW Machaquilá, 16.44661°, −89.54939° ±8m, 400m, 27–30 May 2009 (LLAMA#Ma-B-06-1-02); 4.5km WNW Machaquilá, 16.40112°, −89.48697° ±13m, 415m, 27–30 May 2009 (LLAMA#Ma-B-06-2-02); *Sacatepéquez*: 5km SE Antigua, 14.53725°, −90.69475° ±4m, 2125m, 10–13 June 2009 (LLAMA#Ma-B-08-1-01); 5km SE Antigua, 14.53650°, −90.69483° ±4m, 2145m, 10–13 June 2009 (LLAMA#Ma-B-08-1-02); 5km SE Antigua, 14.52846°, −90.68874° ±6m, 2335m, 10–13 June 2009 (LLAMA#Ma-B-08-2-01); *Suchitepéquez*: 4km S Vol. Atitlán, 14.54804°, −91.19108° ±7m, 1580m, 15–18 June 2009 (LLAMA#Ma-B-09-1-01); 4km S Vol. Atitlán, 14.54807°, −91.19188° ±4m, 1575m, 14–18 June 2009 (LLAMA#Ma-B-09-1-02); 4km S Vol. Atitlán, 14.54852°, −91.19331° ±7m, 1590m, 14–18 June 2009 (LLAMA#Ma-B-09-2-01); HONDURAS, *Olancho*: PN La Muralla, 15.09490°, −86.73987° ±10m, 1410m, 2–5 May 2010 (LLAMA#Ma-C-01-2-02); PN La Muralla, 15.09721°, −86.73840° ±30m, 1480m, 2–5 May 2010 (LLAMA#Ma-C-01-3-01); *Comayagua*: PN Cerro Azul Meambar, 14.86987°, −87.89885° ±10m, 1150m, 20–23 May 2010 (LLAMA#Ma-C-04-2-01); *Cortés*: PN Cusuco, 15.48898°, −88.23707° ±10m, 1260m, 30 May–3 June 2010 (LLAMA#Ma-C-06-1-01); PN Cusuco, 15.48839°, −88.23592° ±10m, 1260m, 30 May–3 June 2010 (LLAMA#Ma-C-06-1-02). COSTA RICA, *Guanacaste*: Santa Rosa Nat. Park, 10.85°, −85.62° ±2km, 300m, 21 February 2003 (*J. S. Noyes*) [JTLC000004338].

*Measurements* (*9 measured*): HW 0.25–0.31, HL 0.18–0.24, EL 0.09–0.12, SL 0.09–0.012, LAII 0.05–0.07, LAIII 0.04–0.06, LAIV 0.04–0.07, LAXIII 0.10–0.14, MH 0.23–0.37, ML 0.38–0.54, PrW 0.18–0.26, PW 0.07–0.10, PL 0.07–0.10, AIIIW 0.16–0.21, AIIIL 0.07–0.12, AIVW 0.15–0.23, AIVL 0.10–0.12, FFeW 0.04–0.06, FFeL 0.20–0.29, HFeL 0.22–0.30, HTiL 0.23–0.34, CI 115–130, PI 82–121, MI 60–71.

##### Description.

 Headbroader than long, with large convex eyes that occupy almost half of the sides of head. Mandible slender and falcate with blunt apex, without differentiated masticatory margin, edentate. External margin of mandible more or less evenly curved along its length. Mandible tips crossing at closure, mandible longer than eye length. Lateroclypeal teeth and hypostomal teeth lacking, clypeus short and transverse, without visible clypeal lamella (apron). Antennal sockets horizontal and exposed, located at the anterior clypeal margin that is projecting anteriorly beyond ventral articulation with labrum. Antenna 13-segmented, each segment longer than wide, with second, third and fourth segments subequal in length. Scape of moderate length, subequal to the length of ultimate antennal segment. Scape about twice the length of the second antennal segment, and about the combined length of the second and third antennal segments. Lateral ocellus separated from median ocellus by little more than its diameter. Distance between lateral ocelli little greater than between median and lateral ocellus and ocelli forming almost equilateral triangle. Mesosomawith distinctive pronotum: U-shaped in dorsal view and reduced anteromedially to a thin horizontal strip, set below the level of the dorsally protruding mesonotum and triangular in lateral view, with pointed posterior apex directed towards the wing base. Mesoscutum lacking notauli and parapsidal lines not discernable. Axillae depressed, not meeting medially, connected by a narrow furrow. Tegula very small and inconspicuous. Mesopleuron lacking oblique transverse sulcus and hence not divided into anepisternum and katepisternum. Mesoscutellum prominently bulging, as seen in lateral view. Metapleural gland not discernable. Propodeum with dorsal and declivous surfaces not differentiated, evenly rounded. Propodeal spiracle small, circular, positioned slightly below midheight of propodeum and slightly posterior to the midlength. Legs slender, mesotibia and metatibia each with two simple spurs, pretarsal claw lacking preapical tooth. Wingwith extremely reduced venation. Fore wing with C present, tubular and weakly pigmented. Sc+R very closely approximated to the wing margin, very narrow, compressed vertically, the most apparent vein on forewing. Sc+R1 region not differentiated in absence of Rs·f1 but differing from rest of vein by not being conspicuously vertically compressed; in line with Sc+R, nebulous. Pterostigma not marked. R1·f3 absent. M+Cu nebulous and inconspicuous, slightly curved towards posterior wing margin before division. Rs·f1 absent. M·f1, Rs+M, Rs·f2 and Rs·f3 all joined, not differentiated, tubular or partially nebulous. 1r-rs absent. 2r-rs present, spectral. Rs·f4 and Rs·f5 joined and not differentiated in the absence of 2rs-m. Rs·f4&f5 nebulous and poorly visible, terminating before wing margin. Free abscissae of M absent. Abscissae of Cu joined, initially nebulous, continuing throughout most of the length as spectral. Vein A tubular, joining cu-a at a very obtuse angle and confluent with Rs+M, apparently absent beyond cu-a, although weak flexion at the posterior wing margin can be interpreted as spectral A·f2&f3. Posterior margin of fore wing with narrow, conspicuous fold where hamuli attach. Hind wing with C present, tubular, reaching about fourth of wing length. Anterior margin of hind wing past midlength with a conspicuous dark stigma. Two hamuli originate in the region of the stigma. Jugal lobe absent. Metasoma slender in lateral view, obovate in dorsal view, widest at abdominal segment IV. Petiole (abdominal segment II) subquadrate in lateral view, about as long as high or wide, and only weakly constricted posteriorly, the helcium thus apparently quite broad. Petiolar spiracle located on anterior third of the segment, near anterodorsal extremity; abdominal segment III larger than petiole, and not developed as postpetiole nor separated from abdominal segment IV by a marked constriction. Abdominal spiracle III located on anterior third of tergite. Petiole and abdominal segment III with tergosternal fusion. Abdominal segment IV and succeeding segments lacking tergosternal fusion. Segment IV with weakly differentiated presclerites. Spiracle present on anterior third of tergite IV. Abdominal segments V and VI lacking well differentiated presclerites, and not separated from succeeding segments by constrictions. Abdominal spiracles V and VI not discernable in specimens examined but possibly present at anterior margins of respective tergites. Abdominal tergite VIII (pygidium) small and simple but visible dorsally, not wholly covered by abdominal tergite VII. Pygostyli absent. Abdominal sternite IX (subgenital plate) with posterior margin broadly and deeply concave but not bifurcate. Basal ring present, not hypertrophied. Paramere small and slender with pointed, slightly outcurved apex of harpago. Paramere little longer than petiole length. Volsella a simple, narrow and elongate lobe, lacking differentiated cuspis, distally pointed and slightly outcurved. Aedeagus little longer than paramere and volsella, simple, narrow, distally spatulate. Body size very small. Integument mostly smooth and shiny, with scattered piligerous punctures. Pilosity common on most of body, suberect to decumbent. Color light yellowish-brown, head and posterior margins of abdominal segments IV–VII darker, appendages (antennae, mandibles, legs) lighter.

**Figure 6.**
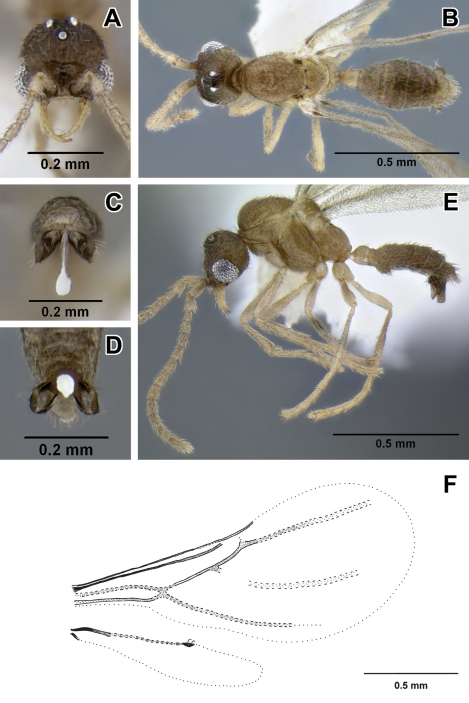
Leptanilloidinae male 1 **A–D** male (CASENT0234558) **A** head in full-face view **B** body in dorsal view **C** genitalia in posterior view **D** genitalia in ventral view **E** male (CASENT0234561) body in lateral view **F** wing venation.

##### Discussion.

 Project LLAMA (Leaf Litter Arthropods of MesoAmerica) is an arthropod biodiversity inventory project carrying out a structured sampling program at sites from southern Mexico (Chiapas) to Nicaragua. The focus is on mature mesophyll forest at multiple elevations. Four days are spent sampling at each study site, and one of the methods is to erect four Malaise traps for the four days. Sampling has been carried out in May and June, 2008 to 2010. The diminutive *Leptanilloides* male described here is surprisingly common in the Malaise samples, occurring at many of the study sites and across a great range of elevations (these specimens temporarily reside in the personal collections of Borowiec and Longino, ultimately to be deposited in major institutional collections). They have been found in the Sierra de Chiapas (near the type locality of *Leptanilloides gracilis*), in the lower elevation Lacandón rainforests of northern Chiapas, in the Petén region of Guatemala, in both lowland and montane regions of central Guatemala, and in montane regions of Honduras. When they occur at a site, they are typically found in more than one of the Malaise traps, but usually no more than about five males per trap in a 4-day sample. They are very easily overlooked because of their similarity, in both size and degree of sclerotization, to small nematoceran Diptera that are often abundant in Malaise samples.

From hitherto described males of *Leptanilloides* (Donoso et al. 2006, Ward 2007, Ward & Brady 2009, this study) and *Amyrmex* (Ward & Brady 2009) this morphospecies can be differentiated by a combination of falcate mandibles, small size and extremely reduced wing venation with Rs·f1 and pterostigma absent in fore wing and hind wing venation restricted to a short C stub, as well as the external structure of the genitalia. The falcate mandibles are similar in shape to mandibles of males of *Leptanilloides nubecula*, but the specimens of male 1 are smaller than the males of *Leptanilloides nubecula* and apparently have less well developed wing venation. Although Donoso et al. (2006) did not describe wing venation in detail for *Leptanilloides nubecula*, and we have not examined the male specimens of that species, from the picture given in their treatment (Fig. 26, p. 55) it is clear that the wing venation is much better developed in *Leptanilloides nubecula*.Rs·f1 can be seen in fore wing and hind wing has a conspicuous Sc+R running almost three fourths of the wing length, while in male 1 both these veins are apparently absent.

The largely sympatric distribution, two simple spurs on mid and hind tibia, overall small size, and relative abundance make these specimens good candidates for the male caste of *Leptanilloides gracilis*.

#### Leptanilloidinae male 2

Figure 7A–F

##### Material examined.

 COSTA RICA, *Puntarenas*: 5km S San Vito, 8.78333°, −82.96667° ±2km, 1200m, 22–26 August 2010, montane wet forest, ex pan trap (*M. Pollet & A. De Braekeleer*) [MLBC].

*Measurements in mm and indices* (*2 measured*): HW 0.37–0.40, HL 0.30–0.31, EL 0.14–0.15, SL 0.13–0.14, LAII 0.07–0.08, LAIII 0.07, LAIV 0.06–0.07, LAXIII 0.14–0.15, MH 0.36–0.41, ML 0.65–0.68, PrW 0.31–0.33, PW 0.10–0.11, PL 0.16, AIIIW 0.19–0.20, AIIIL 0.15–0.19, AIVW 0.28–0.29, AIVL 0.20–0.22, FFeW 0.08, FFeL 0.32–0.36, HFeL 0.36–0.38, HTiL 0.37–0.40, CI 125–127, PI 65–67, MI 56–59.

##### Description.

 Headbroader than long, with large convex eyes that occupy almost half of the sides of head. Mandible slender, tapering to pointed apex, without differentiated masticatory margin, edentate. External margin of mandible more or less straight along its length. Mandible tips crossing at closure, mandible slightly longer than eye length. Lateroclypeal teeth and hypostomal teeth lacking, clypeus short and transverse, without visible clypeal lamella (apron). Antennal sockets horizontal and exposed, located at the anterior clypeal margin that is not projecting anteriorly beyond ventral articulation with labrum. Antenna 13-segmented, each segment longer than wide, with second, third and fourth segments subequal in length. Scape of moderate length, subequal to the length of ultimate antennal segment. Scape length about twice the length of the second antennal segment, and about the combined length of the second and third antennal segments. Lateral ocellus separated from median ocellus by little more than its diameter. Distance greater between lateral ocelli than between median and lateral ocellus and ocelli forming isosceles triangle. Mesosomawith distinctive pronotum: U-shaped in dorsal view and reduced anteromedially to a thin horizontal strip, set below the level of the dorsally protruding mesonotum and triangular in lateral view, with pointed posterior apex directed towards the wing base. Mesoscutum lacking notauli and parapsidal lines present, weakly marked but long, running about two thirds of mesoscutum length. Axillae depressed, not meeting medially, connected by a narrow furrow; tegula very small and inconspicuous. Mesopleuron lacking oblique transverse sulcus and hence not divided into anepisternum and katepisternum. Mesoscutellum raised above level of mesosctum but not prominently bulging, as seen in lateral view. Metapleural gland not discernable. Propodeum with dorsal surface clearly shorter than declivous. Propodeal spiracle small, circular, positioned at midheight of propodeum and slightly posterior to the metanotum. Legs slender, mid tibia with one simple and hind tibia with one pectinate spur, pretarsal claw lacking preapical tooth. Wingwith relatively well developed venation (for *Leptanilloides*). Fore wing with C present, tubular and weakly pigmented. Sc+R very closely approximated to the wing margin, very narrow, compressed vertically. Sc+R1 region joining Sc+R at obtuse angle, tubular. Pterostigma well marked. R1·f3 absent. M+Cu nebulous but conspicuous, slightly curved towards posterior wing margin before division. Rs·f1 stub present, tubular but not reaching Sc+R. M·f1 pigmented, tubular. Rs+M tubular and pigmented, straight. Rs·f2 and Rs·f3 joined, not differentiated, tubular and pigmented. 1r-rs absent. 2r-rs present, tubular and pigmented. Rs·f4 and Rs·f5 joined and not differentiated in the absence of 2rs-m. Rs·f4&f5 partly tubular and partly nebulous, terminating before wing margin. Free abscissae of M present, nebulous and very weakly visible. Abscissae of Cu joined, nebulous throughout most of the length and continuing as spectral. Vein A tubular, joining cu-a at obtuse angle and confluent with Rs+M, apparently absent beyond cu-a. Posterior margin of fore wing with narrow, conspicuous fold where hamuli attach. Hind wing with C absent. Rc+R present, tubular but compressed, reaching about third of wing length. Anterior margin of hind wing with little differentiated pigmentation. Three hamuli originate in the pigmented region. Jugal lobe absent. Metasoma slender in lateral view, obovate in dorsal view, widest at abdominal segment IV. Petiole (abdominal segment II) ovate in lateral view, longer than high or wide, and weakly constricted posteriorly, the helcium thus apparently quite broad. Petiolar spiracle located on anterior third of the segment, near anterodorsal extremity. Abdominal segment III larger than petiole, and not developed as postpetiole nor separated from abdominal segment IV by a marked constriction. Abdominal spiracle III located on anterior third of tergite. Abdominal segments II and III with tergosternal fusion. Abdominal segment IV and succeeding segments lacking tergosternal fusion. Segment IV with weakly differentiated presclerites. Spiracle present on anterior third of tergite IV. Abdominal segments V and VI lacking well differentiated presclerites, and not separated from succeeding segments by constrictions. Abdominal spiracles V and VI not discernable in specimens examined but possibly present at anterior margins of respective tergites. Pygostyli absent. Abdominal sternite IX (subgenital plate) was hidden and not observed. Basal ring present, not hypertrophied. Paramere relatively broad, not tapering, apically harpago truncated. Paramere little longer than petiole length. Volsella simple, lacking differentiated cuspis, tapering suddenly at midlength and distally pointed, forming ventrally directed hooks. Aedeagus apparently very short, could not be observed directly without dissection. Body size moderate. Integument mostly smooth and shiny, with scattered piligerous punctures. Pilosity common on most of body, suberect to decumbent. Color light brown, head, and mesoscutellum darker. Antennal segments I–III light, the rest light brown. Other appendages (mandibles, legs) lighter.

**Figure 7.**
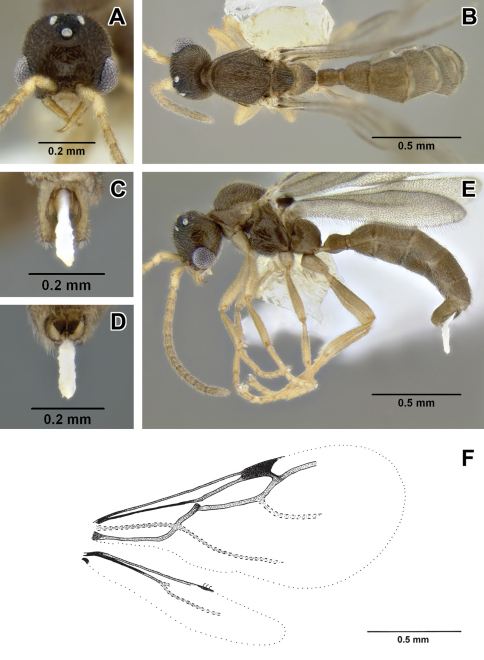
Leptanilloidinae male 2 **A–E** male (CASENT0234556) **A** head in full-face view **B** body in dorsal view **C** genitalia in posterior view **D** genitalia in ventral view **E** body in lateral view **F** wing venation.

##### Discussion.

 These two males are from 1200m elevation wet forest, at the Wilson Botanical Garden in southern Costa Rica. They were collected by Marc Pollet in yellow pan traps on the forest floor, in late August, 2010.

These large male specimens can be recognized by sublinear, evenly tapering mandible without differentiated basal and masticatory margins, moderate size and relatively well developed wing venation. From Leptanilloidinae male 3 they differ in subequal dorsal and declivous faces of propodeum (dorsal surface shorter in male 3), shorter petiole and free abscissae of M joining Rs+M. From *Leptanilloides mckennae* they can be distinguished by arched propodeum (flattened in *mckennae*) and sublinear mandibles (subtriangular in *mckennae*).

#### Leptanilloidinae male 3

Figure 8A–F

##### Material examined.

 MEXICO, *Chiapas*: Lago Metzabok, 17.12681°, −91.63094° ±6m, 570m, 5–8 June 2008 (LLAMA#Ma-A-06-1-02); GUATEMALA, *Petén*: Cerro Cahuí, 17.00044°, −89.70346° ±5m, 140m, 22–25 May 2009 (LLAMA#Ma-B-05-1-02); 4.5km WNW Machaquilá, 16.40112°, −89.48697° ±13m, 415m, 27–30 May 2009 (LLAMA#Ma-B-06-2-02).

*Measurements in mm and indices* (*3 measured*): HW 0.42–0.43, HL 0.32–0.33, EL 0.16, SL 0.13–0.14, LAII 0.08–0.09, LAIII 0.07–0.09, LAIV 0.10–0.11, LAXIII 0.17–0.19, MH 0.45–0.48, ML 0.66–0.70, PrW 0.32–0.35, PW 0.08–0.10, PL 0.18–0.20, AIIIW 0.20–0.25, AIIIL 0.20–0.25, AIVW 0.25–0.34, AIVL 0.20, FFeW 0.06–0.07, FFeL 0.37, HFeL 0.41–0.43, HTiL 0.40–0.43, CI 127–136, PI 41–54, MI 65–72.

##### Description.

 Headbroader than long, with large convex eyes that occupy almost half of the sides of head. Mandible slender, widest at midlength but without differentiated masticatory margin, tapering to pointed apex, edentate. External margin of mandible more or less straight along its length. Mandible tips crossing at closure, mandible length subequal to eye length. Lateroclypeal teeth and hypostomal teeth lacking, clypeus short and transverse, with narrow clypeal lamella (apron). Antennal sockets horizontal and exposed, located at the anterior clypeal margin that is not projecting anteriorly beyond ventral articulation with labrum. Antenna 13-segmented, each segment longer than wide, with third segment the shortest. Scape of moderate length, subequal to the length of ultimate antennal segment. Scape length less than twice the length of the second antennal segment, and less than the combined length of the second and third antennal segments. Lateral ocellus separated from median ocellus by more than its diameter. Distance greater between lateral ocelli than between median and lateral ocellus and ocelli forming isosceles triangle. Mesosomawith distinctive pronotum: U-shaped in dorsal view and reduced anteromedially to a thin horizontal strip, set below the level of the dorsally protruding mesonotum and triangular in lateral view, with pointed posterior apex directed towards the wing base. Mesoscutum lacking notauli. Parapsidal lines present, long, running about the third of mesoscutum length. Axillae depressed, not meeting medially, connected by a narrow furrow; tegula very small and inconspicuous. Mesopleuron lacking oblique transverse sulcus and hence not divided into anepisternum and katepisternum. Mesoscutellum raised above level of mesoscutum and prominently bulging, as seen in lateral view. Metapleural gland not discernable. Propodeum with dorsal surface somewhat shorter than declivous. Propodeal spiracle small, circular, positioned slightly above midheight of propodeum and slightly posterior to the metanotum. Legs slender, mid tibia with one simple and hind tibia with one pectinate spur, pretarsal claw lacking preapical tooth. Wingwith relatively well developed venation. Fore wing with C present, tubular and pigmented. Sc+R approximated to the wing margin, very narrow, compressed vertically. Sc+R1in line with Sc+R, tubular. Pterostigma well marked. R1·f3 absent. M+Cu tubular, slightly curved towards posterior wing margin before division. Rs·f1 present, nebulous. M·f1 pigmented, tubular. Rs+M&Rs·f2&Rs·f3 tubular and pigmented. 1r-rs absent. 2r-rs present, tubular and pigmented. Rs·f4&Rs·f5 tubular, terminating before wing margin. Free abscissae of M nebulous, very weakly visible and not joining to Rs+M&Rs·f2&Rs·f3. Abscissae of Cu joined, nebulous throughout most of the length and continuing as spectral. Vein A tubular, joining cu-a at obtuse angle and confluent with Rs+M, apparently absent beyond cu-a. Posterior margin of fore wing with fold where hamuli attach narrow, conspicuous. Hind wing with C apparently present, narrow and faint except basal fourth of wing length. Sc+R present, tubular along fourth of wing length, continuing as nebulous. Sc+R1 a short nebulous stub. Rs·f1&Rs·f2 nebulous, terminating at about three fourth of wing length. Anterior margin of hind wing with little differentiated pigmentation. Three hamuli originate in the pigmented region. Jugal lobe absent. Metasoma slender in lateral view, obovate in dorsal view, widest at abdominal segment IV. Petiole (abdominal segment II) elongate-ovate in lateral view, more than two times longer than high or wide, and weakly constricted posteriorly, the helcium thus apparently quite broad. Petiolar spiracle located on anterior fourth of the segment, near anterodorsal extremity. Abdominal segment III larger than petiole, and not developed as postpetiole nor separated from abdominal segment IV by marked constriction. Abdominal spiracle III located on anterior third of tergite. Abdominal segments II and III with tergosternal fusion. Abdominal segment IV and succeeding segments lacking tergosternal fusion. Segment IV with weakly differentiated presclerites. Spiracle present on anterior third of tergite IV. Abdominal segments V and VI lacking well differentiated presclerites, and not separated from succeeding segments by constrictions. Abdominal spiracles V and VI not discernable in specimens examined but possibly present at anterior margins of respective tergites. Abdominal tergite VIII (pygidium) small and simple but visible dorsally, not wholly covered by abdominal tergite VII. Pygostyli absent. Abdominal sternite IX (subgenital plate) with posterior margin broadly and deeply concave but not bifurcate. Basal ring present, not hypertrophied. Paramere relatively broad, harpago evenly rounded at apex; paramere shorter than petiole length. Volsella a simple, broad and elongate lobe, lacking differentiated cuspis, distally pointed. Aedeagus about equal in length to paramere and volsella, simple, narrow, distally spatulate. Body size moderate. Integument mostly smooth and shiny, with scattered piligerous punctures. Pilosity common on most of body, suberect to decumbent. Color light brown, head and metasoma past abdominal segment III darker. Antennal segment II light, the rest light brownish. Other appendages (mandibles, legs) lighter than body.

**Figure 8.**
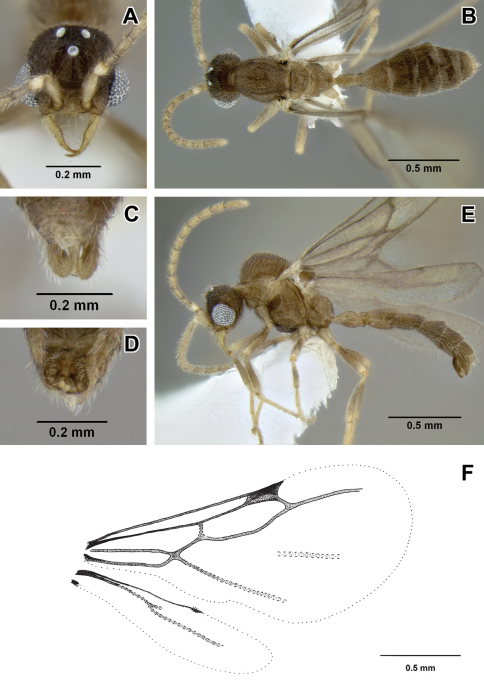
Leptanilloidinae male 3 **A–E** male (CASENT0617071) **A** head in full-face view **B** body in dorsal view **C** genitalia in posterior view **D** genitalia in ventral view **E** body in lateral view **F** wing venation.

##### Discussion.

 This form has been collected at two sites in the Petén region of Guatemala and one locality in Chiapas, Mexico.

This relatively large male differs from Leptanilloidinae male 2 and *Leptanilloides mckennae* in the dorsal face of the propodeum being shorter than the declivity (subequal in male 2 and flattened in *mckennae*), longer petiole, and free abscissae of M not connected to Rs+M. Additionally, from *mckennae* it differs by the slender mandibles without well differentiated masticatory and basal margins (subtriangular in *mckennae*). We have examined an additional specimen from Barro Colorado Island, Panama (“Leptanilloidine genus 1 PM01”; CASENT0106194), already mentioned by Ward & Brady (2009) that may belong here. It is larger (ML 0.74) with wider head (HW 0.43) and larger eyes (EL 0.20) but with relatively shorter petiole (PW 0.10, PL 0.15). The wing venation is similar, except veins of radial sector being more approximated to the anterior wing margin and thus making the closed veins of the wing appear more flattened. There is also a stub of free abscissae of M, completely absent in the three males from Mexico and Guatemala. Genitalia in this specimen are retracted and partly obscured, but seem similar to the genitalia present in Leptanilloidinae male 3. In the absence of collections of males of similar morphotypes between Guatemala and Panama, we are unable to tell whether this form represents a geographical variant or a distinct species.

## Conclusions

The leptanilloidine ants, apparently due to their presumably subterranean habits, represent a serious challenge to sampling. The ratio of collecting events to number of worker-based morphospecies continues to be high, and number of male morphotypes present in the collections from recent efforts in Central America (LLAMA project) exceeds the number of the known worker-based species from the same region. This makes it certain that new species will continue to be discovered. When molecular data become available for more workers and unassociated males of *Leptanilloides* and workers of *Asphinctanilloides*, it seems most probable that one of the genera is identical to *Amyrmex*. Given the unsatisfactory state of knowledge of the subfamily, future efforts documenting the diversity, biology, morphology, internal phylogeny, as well as phylogenetic position of Leptanilloidinae within dorylomorphs are much needed.

## Supplementary Material

XML Treatment for Leptanilloides erinys

XML Treatment for Leptanilloides femoralis

XML Treatment for Leptanilloides gracilis

XML Treatment for Leptanilloidinae

XML Treatment for Leptanilloidinae

XML Treatment for Leptanilloidinae
